# Risk Factors for Testicular Cancer: Environment, Genes and Infections—Is It All?

**DOI:** 10.3390/medicina59040724

**Published:** 2023-04-07

**Authors:** Sertac Yazici, Dario Del Biondo, Giorgio Napodano, Marco Grillo, Francesco Paolo Calace, Domenico Prezioso, Felice Crocetto, Biagio Barone

**Affiliations:** 1 Department of Urology, Hacettepe University School of Medicine, 06230 Ankara, Turkey; 2 Department of Urology, ASL NA1 Centro Ospedale del Mare, 80147 Naples, Italy; 3 University of Rome Tor Vergata, 00133 Rome, Italy; 4 Department of Neurosciences, Reproductive Sciences and Odontostomatology, University of Naples “Federico II”, 80131 Naples, Italydprezioso@libero.it (D.P.);

**Keywords:** testicular cancer, risk factor, genetics, infections, environment, andrology, urology

## Abstract

The incidence of testicular cancer is steadily increasing over the past several decades in different developed countries. If on one side better diagnosis and treatment have shone a light on this disease, on the other side, differently from other malignant diseases, few risk factors have been identified. The reasons for the increase in testicular cancer are however unknown while risk factors are still poorly understood. Several studies have suggested that exposure to various factors in adolescence as well as in adulthood could be linked to the development of testicular cancer. Nevertheless, the role of environment, infections, and occupational exposure are undoubtedly associated with an increase or a decrease in this risk. The aim of this narrative review is to summarize the most recent evidence regarding the risk factors associated with testicular cancer, starting from the most commonly evaluated (cryptorchidism, family history, infections) to the newer identified and hypothesized risk factors.

## 1. Introduction

Testicular cancer is the most common type of neoplasm among young men aged 15 to 40 years, representing 1% of adult neoplasms and 5% of urological tumors, reaching an incidence of 3–11 cases per 100,000 males per year in the Western world [1]. Although it is relatively rare, testicular cancer is an important public health concern due to its impact on the quality of life and reproductive function of affected men [2]. Early detection and treatment of testicular cancer are crucial to improve outcomes and death risk reduction [3]. In particular, testicular cancer represents a sort of unicity among cancers due to the possibility to be effectively treated with surgery and/or radiation therapy and presenting an overall excellent prognosis, with a >90% cure rate and >95% five-year survival rate, especially for early detected patients [4]. However, considering the lack of clear symptoms and signs in the early stages, with the exception of a unilateral lump or painless swelling, the early detection of the disease could be challenging [5]. This is the reason why understanding the risk factors associated with testicular cancer is crucial for early detection and treatment. The exact causes of testicular cancer are not well understood, but several risk factors have been identified, including age, cryptorchidism, abnormal testicle development, personal and familiar history of testicular cancer, ethnicity, and a weakened immune system [6]. Additionally, certain medical conditions, such as Klinefelter syndrome and Down syndrome, may also increase the risk of developing testicular cancer [7,8,9]. Considering the rarity of the condition, the relative ease of diagnosis due to the accessibility of the testis to imaging and clinical exploration, and the potential impairment to fertility related to the treatment, the role and the identification of risk factors in the development of testicular cancer still represent a topic of high clinical interest. The aim of this research paper is, therefore, to examine and summarize the current state of knowledge regarding the risk factors for this malignancy, including demographic and environmental factors, lifestyle factors, and medical conditions, posing particular attention to the most recent evidence and hypotheses.

## 2. Epidemiology and Global Trends

Although testicular cancer represents a relatively rare disease, accounting for about 1% of cancers and ranking 26th for cancer incidence in men, the estimated overall incidence worldwide in 2020 was 74,458 cases with an age-standardized rate of 1.8:100,000 among all cancers, showing an increase of over 1.80-fold in the last 25 years [10]. In particular, when geographical areas are considered, Asia, Europe, and Northern America reported the highest estimated incidence rate with, respectively, 5,021,195, 2,339,617, and 1,372,002 cases, for an ASR of 185.2, 328.5, and 397.9:100,000. Regarding mortality, testicular cancer reported a total of 9334 estimated deaths worldwide, with an ASR of 0.22:100,000, mostly distributed among Asia (3997 deaths, ASR 0.16:100,000), Latin America (2139 deaths, ASR 0.60:100,000), and Europe (1568 deaths, ASR 0.34:100,000) [11,12]. The global pattern of testicular cancer incidence and mortality is however highly heterogeneous, with a higher incidence rate in high human development index (HDI) countries and, conversely, a higher mortality in low HDI countries. This data is consistent with adequate diagnosis and treatment protocols implemented in the highest-income countries since the 1970s, associated with reduced exposures to well-known risk factors such as cryptorchidism and pharmaceuticals during pregnancy [13,14]. Conversely, in lower HDI countries such as Latin America, testicular cancer mortality rates have increased in parallel to incidence due to inequitable distribution of specialists and centers and difficulties in accessing cancer care for rural and remote populations [15,16].

## 3. Etiology and Histopathology

Testicular cancer could derive from any cell type found in the testicles. Nevertheless, more than 95% of testicular cancers arise from germ cells, which are further divided, according to the histologic features, into seminomas, non-seminomas, and spermatocytic seminomas. The remaining 5% is represented by sex cord or stromal cell tumors as well as miscellaneous non-specific stromal cell tumors [17]. Interestingly, the incidence of testicular cancer does not increase with age but instead presents a peak at 25–29 years for non-seminomas and 35–39 for seminomas [18,19]. Despite several environmental and hormonal factors that have been hypothesized to be related to testis carcinogenesis, nevertheless, the only factors clearly associated with testicular cancer are prior unilateral testicular cancer, family history of testicular cancer, and congenital anomalies such as cryptorchidism [20,21,22]. Germ cell tumors develop secondary to a tumorigenic event in utero, successively leading to an intratubular germ cell neoplasia, which is derived from gonocytes that failed to differentiate into spermatogonia. With the hormonal changes happening during puberty, these cells develop their invasive potential. Seminomas consist instead of transformed germ cells with a blocked differentiation. Finally, embryonal carcinoma cells are more similar to undifferentiated stem cells with a gene expression similar to those cells and intratubular germ cell malignancies. Stromal cell tumors, as well as sex cord and non-specific stromal tumors, have instead an extraembryonic/somatic differentiation [18,23].

Testicular cancer definitions are based on cell type derivation. According to the WHO, the histopathological classification comprises [24]:

1. Germ cell tumors.
2. Derived from Germ Cell neoplasia in situ (GCNIS): seminoma, embryonal carcinoma, yolk sac tumor (post-pubertal), trophoblastic tumors, teratoma, mixed germ cell tumors.
3. Germ cell tumors unrelated to GCNIS: spermatocytic tumor, yolk sac tumor (pre-pubertal), mixed germ cell tumor (pre-pubertal).
4. Sex cord/stromal: Leydig cell tumor, Sertoli cell tumor, granulosa cell tumor, thecoma, unclassified, gonadoblastoma.
5. Miscellaneous non-specific tumors: ovarian epithelial tumors, tumors of the collecting ducts and rete testis, adenoma, carcinoma, adenomatoid tumor, mesothelioma, epididymal tumor, cystadenoma of the epididymis, papillary cystadenoma, mesenchymal tumor of the spermatic cord.

## 4. Epidemiological Risk Factors

### 4.1. Cryptorchidism

Cryptorchidism is the most common birth defect involving male genitalia and is characterized by the absence of at least one testicle from the scrotum, most commonly the right. Up to 80% of cryptorchid testes descend within the third month of life [25]. Nevertheless, about 10% of all cases of germ cell tumors occur in men with a history of cryptorchidism with the most accredited hypothesis related to the elevated temperature of the undescended testis, thus inhibiting the differentiation of spermatogonia and resulting in an arrest of spermatogenesis, germ cell depletion, and fibrosis. In addition, the altered position of the testis could alter the function of the somatic cells forming the niche for spermatogonial stem cells’ self-renewal and differentiation. The overall risk of developing testicular cancer in patients who were or are cryptorchid is 3.7–7.5 times higher than in the normal population [26,27]. Although corrective surgery diminishes this risk by half, the former cryptorchid testis becoming cancerous indicates that permanent epigenetic changes are reported in the testis [28]. Recent evidence suggests that allelic variants in genes implicated in the development of the testes could be present in a patient with cryptorchidism. In particular, *KIT* gene variants might be the determinants in the association between this condition and testicular cancer [29]. However, up to now, even if an irrefutable higher risk of testicular cancer in patients affected by previous or current cryptorchidism has been found, the real pathogenetic mechanism underlying this association is still unclear [30].

### 4.2. Family History

Based on clinical observations and systematic investigations, it has been suggested that a family history of testicular cancer represents a major risk factor for this kind of cancer. In particular, it has been evaluated that there is a 3.1-fold increased relative risk for first-degree relatives of patients with testicular cancer, despite the fact that the age at presentation was not significantly different compared to patients without a familiar history of this cancer [31]. A proper familiar testicular cancer, i.e., two or more affected men in the same bloodline, is quite uncommon, although it has been estimated to occur in about 3% of families. Nevertheless, considering the rarity of the condition at the baseline, a genetic analysis is difficult and even when it has been performed, no cytogenetic abnormalities were found [32,33,34]. A prospective study involving about two million males born from 1951 to 2015 reported 2686 cases, highlighting a 6.3-fold risk for brothers of patients affected by testicular cancer, 4.7-fold for sons, 4.4-fold for fathers, 2.0-fold for paternal uncles, and 1.9-fold for maternal uncles. Interestingly, an increased risk for testicular cancer was found also for patients with a family history of mesothelioma (4.4 to 2.8-fold), malignant melanoma (1.4-fold), and malignant neuroepithelial tumors (11.1 to 4.6-fold), suggesting, in those cases, the existence of hereditary cancer syndromes [35]. Several other previous linkage studies reported similar conclusions, adding interesting data such as a higher prevalence of cryptorchidism and a younger age at diagnosis among cousins pairs; a similar age of testicular cancer development among relatives; a potential role of shared childhood environment [36,37,38].

### 4.3. Maternal and Perinatal Factors

Considering the natural history of testicular cancer and the relatively young age at the diagnosis, exposure to risk factors during early life could be a part of the initial stages of carcinogenic transformation. Cryptorchidism represents the most obvious example. In particular, a recent meta-analysis suggested how low birth weight, perinatal inguinal hernia, and twinning, in addition to cryptorchidism, are associated with an increased risk of testicular cancer, reporting, respectively, an odds ratio (OR) of 1.34, 1.63, and 1.22 [39]. Another potential risk factor is associated with the age of the mother at conception. In particular, older ages of mothers at conception were associated with a reduced risk of testicular cancer (OR = 0.73), in addition to a relatively lower risk in men who had been breastfed for 6 months or more (OR = 0.63) [40]. These findings support the potential role of higher estrogen exposure in mothers as a potential risk factor for testicular cancer [41]. Interestingly, to support this evidence, a comprehensive meta-analysis by Hom et al. reported an overall OR = 2.98 for testicular cancer in mothers who were exposed to synthetic estrogen diethylstilbestrol (DES) during pregnancy [42]. Although DES use had been stopped in 1971, it is widely suspected that exposure to other endocrine-disrupting chemicals with estrogenic activity has a role in the carcinogenesis of testes [43].

### 4.4. Age

As previously reported in the epidemiological data, the age distribution of testicular cancer presents a peak at the ages of 25–35 while a smaller peak is reported after 80 years of age. The peculiar age distribution of this cancer is mostly supposedly related to sex hormone activity. Nevertheless, testicular cancer is rare before age 15 [44]. Regarding testicular cancer in older men (>50 years), it has to be reported, however, that the most commonly occurring testicular malignancy is testicular lymphoma, often secondary to non-Hodgkin lymphoma, while the primary testicular lymphoma is rare and could have a different age range [45,46,47].

### 4.5. Ethnicity

Different research posed particular attention to the ethnic differences in testicular cancer, considering that the incidence of this disease largely varies among Caucasians, Hispanics, Asians, and African-Americans. In particular, as reported by Li et al., which analyzed the data of SEER (Surveillance, Epidemiology and End Results), Caucasians reported the highest incidence rate (2.08:100,000), followed by Hispanics (1.19:100,000), Asians (0.60:100,000), and African-Americans (0.36:100,000) [48]. Nevertheless, considering the increasing incidence in the last years also in Hispanics (+2.10% yearly) and Asians (+2.47% yearly), it could be highly possible that environmental exposures or socio-economical disparities could interact synergically with genetic susceptibility [49,50].

### 4.6. Hormonal Levels

Apart from the role of hormones during fetal life, little to no evidence is reported regarding the effects of sex hormones on testicular cancer. A 1997 study by Petridou et al., reported how patients with baldness reported a lower risk of testicular cancer, suggesting indirect evidence of the protective role of androgens regarding testicular cancer [51]. In particular, to support this hypothesis, a previous study by Depue et al. showed how a story of severe acne at puberty was inversely correlated with testicular cancer. Despite the likely relation of androgens to pubertal acne, it has not been conclusively established [52,53]. A more recent meta-analysis by Zhou et al. reported a protective role of baldness against testicular cancer, with an OR = 0.61, however, the work was performed on five case-control studies, thus limiting the potential conclusions [54]. Finally, a study by Nakagawa et al. showed a protective role of the androgen pathway on testicular cancer, suppressing the cell growth of seminomas in vitro and in vivo [55].

### 4.7. Age at Puberty

Several studies have reported a potential link between early puberty and increased risk of testicular cancer, although data are controversial [56]. It is known that patients with precocious puberty are at increased risk of Leydig cell tumors, a rare testicular tumor that could provoke a pseudo precocious puberty [57]. A meta-analysis by Maule et al. involving 12 studies showed a protective role of late puberty (defined as the age of starting shaving), reporting an OR = 0.87 with no effects of early puberty on testicular cancer risk [58].

### 4.8. Body Mass Index

The association between body mass index (BMI) and testicular cancer is also controversial. Although height has been somewhat associated with an increased risk of germ cell testicular cancer in a 2002 study, no significant associations were found regarding BMI and weight. As stated by the authors, this data could be a proxy for energy intake during early life and therefore could be biased [59]. Conversely, a 2006 study by Bjørge et al. reported, on 1357 testicular cancers, a protective role of increased BMI, with overweight and obese men showing an OR of 0.89 and 0.83, respectively. Interestingly, the risk of testicular cancer was not associated with adolescent BMI [60]. Another study by Dieckmann et al. reported, instead, on a total of 8498 testicular germ cell cancer patients, an increased risk for testicular germ cell cancer in young patients with BMI 25 to <30 kg/m^2^ [61]. Finally, a meta-analysis by Lerro et al. reported, on a total of 14 studies, an increased risk of testicular cancer for every 5 cm of height (OR = 1.13), along with a protective role of weight (OR = 0.92), providing support for a positive association between height and testicular cancer and a hypothetical protective role of increased BMI versus this cancer [62].

### 4.9. Infections

The relation between infections and testicular cancer relies mostly on the response to chronic inflammation which is involved in several different steps leading to carcinogenesis. Inflammation cells such as macrophages and leukocytes produce, indeed, reactive oxygen and nitrogen species that could affect DNA integrity [63,64]. One of the first infections identified as a possible risk factor for testicular cancer is represented by HPV, as reported by Garolla et al. In particular, analyzing 155 testicular cancer patients, the prevalence of HPV infection in the semen was 9.5% compared to 2.4% in healthy controls [65]. A large meta-analysis by Trabert et al., involving a total of 767 patients with 929 healthy controls, reported, instead, in an evaluation of different childhood infections as mumps and orchitis, the absence of a certain association between these infections and testicular cancer [66]. Another contemporary meta-analysis by Yousif et al. analyzing EBV, CMV, Parvovirus B19, and HIV, reported an OR for testicular cancer of, respectively, 4.80, 1.85, 2.86, and 1.79, supporting a possible association between these infections and testicular cancer. However, the marked heterogeneity of the studies involved and the relatively small sample size led the authors to consider this association with a certain caution [67]. Interestingly, Kao et al. reported, in patients with a prior diagnosis of epididymoorchitis, a higher prevalence of testicular cancer compared to those without a history of this disease (11% versus 0.3%), with an impressive OR, calculated via a conditional logistic regression analysis, of 38.24 which increased to 47.17 when adjusted for other variables (urbanization level, presence of testicular microlithiasis, and geographical region), highlighting an important role of epididymoorchitis in testicular carcinogenesis. Unfortunately, the limited geographical area (China), the lack of information regarding bacterial culture, and the impossibility to exclude other risk factors such as family history and occupational exposure, limit the results obtained in this study [68]. Finally, a newer and more recent meta-analysis from Garolla et al. reported, on a total of 20 studies with 265,057 patients included, an association between EBV and HIV to testicular cancer, with an OR of, respectively, 7.38 and 1.71 while no association was instead found for HPV, CMV, and Parvovirus B19 [69].

### 4.10. Testicular Trauma

Testicular trauma has been included in the past as a potential risk factor for testicular cancer but its current role in testes carcinogenesis has been diminished [70]. Despite an initial elevated risk for testicular cancer in relation to the testis or groin trauma having been found, data were inconsistent and the hypothesis of an aetiologic role of testis trauma in testicular cancer has not been supported [71]. It seems more plausible that a prior testis trauma could lead the patients to seek proper medical attention and, therefore, lead to the diagnosis of testicular cancer [72,73].

### 4.11. Smoking

Testicular cancer appears to have the least amount of data related to its relationship to cigarette and tobacco smoking. According to a study by Srivastava and Kreiger, a significant association was found between testicular cancer and smoking, in particular for those who smoked 12 to 24 pack-years, reporting an OR of 1.96 which could increase up to 2.31 [74]. Nevertheless, a recent meta-analysis found only a slight relevant association between these two factors, reporting an OR of 1.18 [75]. Even if this association was not fully confirmed, interesting data was found in the study by O’Donnell et al. which reported, in men already diagnosed with testicular cancer, an increased OR of 1.86 of having a large tumor >4 cm in smokers compared to nonsmokers with a significantly increased risk of relapse after therapy (OR = 2.05) [76]. Data regarding passive smoke or during pregnancy yielded controversial results, with no clear association found between the exposure to smoke in utero and the development of testicular cancer [77,78,79].

### 4.12. Drugs

Differently from the exposure to tobacco smoke, the use of recreational drugs and, in particular cannabis, seems to be associated with an increased risk of testicular tumor. Compared to a never-user, the consumption of cannabis yields a twofold increase in developing testicular cancer, with an OR of 1.94 while the use of cocaine was negatively associated with this tumor (OR = 0.54) [80]. A meta-analysis involving three studies highlighted how the current, chronic, and frequent cannabis use was strongly associated with the development of testicular germ cell tumors, reporting an overall OR of 2.49 while no evidence was found regarding the association with seminoma tumors [81]. The rationale underlying this association is, however, still unclear.

### 4.13. Physical Activity

The role of physical activity in testicular cancer represents another controversial factor. As reported by an old study, strenuous physical activity was associated with a moderate effect on the risk of testicular cancer, reporting an OR = 2.36 which increased to 2.58 for strenuous physical activity greater than five times a week [82]. Nevertheless, more recent evidence reported no association with testicular cancer, with a lack of internal consistency of the findings of prior studies [83,84]. As for other risk factors, a meta-analysis including thirteen studies permitted to clear the contrasting findings, reporting, indeed, no evidence of an association between physical activity and subsequent risk of testicular cancer. However, it has to be stated that the observational studies included in the meta-analysis had several limitations which could have affected the results and the heterogeneity of the findings. This lack of association should, indeed, not be interpreted as the non-existence of potential effects of physical activity on testicular cancer risk and further studies should be required [85].

### 4.14. Diet

Diet has been associated with testicular cancer in several older studies, with particular attention to the consumption of milk and dairy products. In a study crossing the data of the International Agency for Research on Cancer (IARC) and the Food and Agriculture Organization (FAO) on 42 countries, cheese was most correlated with the incidence of testicular cancer between 20 and 39 years, followed by animal fats and milk, reporting a correlation coefficient (r) of 0.804 when consumed at prepubertal ages and 0.654 when consumed after puberty [86]. In an effort to evaluate this correlation between dairy products and animal fats and testicular cancer, Walcott et al. conducted a hospital-based case-control study involving 159 patients and 136 matched controls, in order to explore, considering the potential influence of estrogens in testicular cancer, the relationship between dietary phytoestrogens and testicular cancer. Although a U-shaped pattern was observed for coumestrol (a natural organic compound acting as a phytoestrogen) and lignans (a large group of polyphenols found in plants), no consistent data was observed [87]. Bonner et al., in a similar study pattern involving 117 patients and 334 controls, did not report any correlation between diet and testicular cancer, although a significant protective role of vitamin E was reported (OR = 0.51) [88]. Conversely, Garner et al. reported, in a case-control study involving 601 patients and 744 controls, a significantly increased risk of developing testicular cancer in subjects consuming high levels of dairy products (OR = 1.87), red meat (OR = 1.49), and baked products (OR = 1.47) [89]. Similar results were reported in two more recent studies, with adjusted OR of 2.37–2.55 for high consumption of dairy products [90,91].

### 4.15. Heat

The role of testicular temperature and heat in testicular function is well known since the 1960s [92,93]. Starting from this evidence, researchers have investigated the possible association between increased testicular temperature and testicular carcinogenesis. To date, no relationship between these conditions has been found despite a potential role of extreme temperatures in the workplace leading to the hypothesis of a potential association with testicular cancer [94]. Similarly, no association was found between varicocele and testicular cancer [95].

### 4.16. Electromagnetic Fields

The effects of electromagnetic fields on testicular cancer, similar to other minor risk factors, are controversial. If 1990s studies reported a potential increased risk of testicular cancer for subjects professionally exposed to a magnetic field, more recent studies did not report an increased risk of testicular cancer even in subjects working near radar units, radiofrequency emitters, electrical machines, and high-voltage lines [96,97,98,99]. Nevertheless, sporadic case reports and small series have reported cases of testicular cancer in workers exposed to radiofrequency waves [100,101]. Similarly, regarding the use of cellular and cordless telephones, no increased risk of testicular cancer was reported [102].

### 4.17. Occupational Risk Factors

Several occupational studies have investigated potential occupations with increased risk of testicular cancer, highlighting how some occupational exposure could also involve higher exposure to environmental factors for occupational purposes [103]. Plastic-related industries were initially associated with an increased risk for testicular cancer, particularly for those involving the production and manufacturing of polyvinyl chloride (PVC). Nevertheless, although a potential link with PVC was suggested due to the exposure to xenoestrogens–phthalates used in PVC which have estrogenic properties, no association was found [104,105]. Similarly, no association was found for other plastic components such as styrene and urethane [106]. Workers in metalworking industries, notwithstanding a heterogeneous range of occupations which makes it difficult to compare cohorts, reported an increased risk for testicular cancer among furnace workers (standardized incidence ratio [SIR] = 2.30), metal temperers (SIR = 5.85), watchmakers (SIR = 7.52), and precision toolmakers (SIR = 2.15) [107,108]. Similarly, paper workers reported an increased risk for testicular cancer (SIR = 7.4) [109]. Nevertheless, it could be possible that considering the high heterogeneity of workers, the retrospective nature of the studies and the presence of potential confounding factors (such as socio-economic status), the data obtained would be highly biased, albeit the effects of heavy metals and extreme temperatures are well known to alter the functionality of testis [110,111,112].

Among concrete workers, which report an overall increased risk of malignant neoplasm, no evidence was found regarding a potential increased incidence of testicular cancer while a study by Dement et al. reported an increased rate of testicular cancer in carpenters (SIR = 2.48) [113,114]. No association with testicular cancer was instead reported for woodworkers and painters [106,115].

Regarding public safety workers, firefighters reported an increased risk of testicular cancer in two studies, with, respectively, a SIR = 3 in the study by Bates et al. and OR = 4.3 in the study by Stang et al. [116,117]. A more recent meta-analysis by Laroche et al. reported an increased risk of testicular cancer in firefighters with an OR ranging from 1.34 to 2.02 [118]. The possible rationale, even if still unclear, could be related to the exposition of chemical compounds that could act as endocrine-disrupting factors [119]. For what concerns police officers, a positive association was found with testicular cancer (OR = 1.31) which was mostly attributed to hand-held radar [108]. However, in a recent study by Sritharan et al. involving 22,595 police officers, this association was not confirmed as statistically significant [120]. Finally, regarding the investigated risk of testicular cancer in military and related personnel, results were controversial. If, on one side, data revealed a comparable rate of testis malignancy between the military and general population, on the other side, the cases expected in military personnel deployed in the Balkans during 1989–99, as well as the Gulf War, were increased about fourfold [108,121,122,123]. However, it could be possible that an active military lifestyle including operational temporary and long-term deployments could contribute to delayed diagnosis and subsequent treatment, potentially biasing data [124].

Despite a lower prevalence of overall cancer incidence among farmers, pesticides are associated with an increased risk of testicular cancer up to threefold, with a significant exposure-response trend [125,126,127]. In particular, exposure to organochlorine pesticides is associated with an increased risk of testicular cancer (OR = 3.01–3.23) [90]. A more recent study by Lerro et al. re-evaluated this risk, reporting an OR of 1.31 [128]. The rationale underlying the association between pesticides and testicular cancer is related to the endocrine-disrupting activity of these compounds which could influence the risk of testicular cancer both during prenatal and postnatal life [129].

Finally, the relationship between occupational and medical exposure to ionizing radiation and testicular cancer has been denied by a systematic literature review performed by Yousif et al. [67].

## 5. Genetic Risk Factors

Despite the lack of clear evidence supporting the genetic background of testicular cancer and the importance of environmental factors in contributing to the development of testicular cancer, the important and crucial role of genetics in the development and risk of testicular cancer is undeniable. Unfortunately, the lack of reliable studies, mostly limited by the rarity of the condition and, therefore, the difficulty in reaching a large sample size, represent one of the major challenges in unveiling the role of genetics in this disease. Starting from linkage studies, such as that of Crockford et al. which involved 237 pedigreed families with a history of testicular cancer, or that of Nathanson et al. which identified the chromosome Y AZFc region (with a gr/gr deletion) as a testicular cancer risk locus, yielding an OR of 3.2 and 2.1 in familial and sporadic testicular cancers respectively, six regions of interest on chromosomes 2p23, 3p12, 3q26, 12p13-q21, 18q21-q23, and Xq27 were identified as susceptibility loci [33,130]. Other gene mutations associated with testicular cancer are those related to *KRAS* and *KIT*. The first encodes a GTPase that activates, among its downstream target, the MAPK and PI3K-AKT pathways. The hyperactivations of these pathways are however associated with the initiation of tumorigenesis in many cancers [131,132]. The second encodes a tyrosine kinase transmembrane receptor and its mutations are observed in up to 25% of seminoma cases [133]. Differently from other malignancies, testicular cancer is, however, a genetically complex and polygenic disease and multiple risk loci contribute to the testis carcinogenesis [134,135]. Three genome-wide association studies have reported several cancer-risk alleles associated with single nucleotide polymorphism. Rapley et al., Turnbull et al. and Kanetsky et al. revealed other loci associated with testicular cancer related to single nucleotide polymorphism changes affecting *KITLG* (ligand for the tyrosine kinase KIT; OR = 2.69), *SPRY4* (inhibitor of mitogen-activated protein kinase downstream of KITLG-KIT; OR = 1.37), *BAK1* (similarly to the previous one; OR = 1.50), *DMRT1* (involved in gender determination; OR = 1.37), and *TERT* and *ATF7IP* (involved in telomere maintenance; OR = 1.54 and OR = 1.27, respectively) [136,137,138]. More recently, the number of interested loci has reached the number of 44, further highlighting the heterogenic and polygenic characteristics of testicular cancer [139]. Overall, three possible pathogenic mechanisms have been hypothesized starting from the identification of these involved loci. The first one is related to the risk loci associated with the transcriptional regulation of cell development (i.e., *GATA4* and *GATA1*), in particular those involved in the specification and differentiation of postnatal testicular development [140,141,142]. The second one involves the genes *PRDM14*,* SALL4*,* POU5F1*, and *DMRT1* which are related to the germ cell specification, sex determination, and maintenance of pluripotency of embryonic stem cells [137,143,144,145,146]. The third one, lastly, is associated with genes involved in microtubule and chromosomal assemblies such as *TEX14*, *WDR73*, *PMF1*, *CENPE*, and *PCNT* [139,143,147,148,149].

## 6. Testicular Cancer: A Multifactorial Disease

As for other malignancies, testicular cancer represents a particular example of the interaction between genetic and environmental factors. Despite an undeniable genetic predisposition, this disease heavily reflects the exposure to certain environmental factors and occupational exposures (Table 1 and Table 2). Indeed, both infertility and testicular cancer share a common origin during fetal life, with a potential noxa that impairs the function of Sertoli and Leydig cells, altering the proper testis function, from germ line development to hormone production. This condition, which could be associated with hypospadias and cryptorchidism, is commonly defined as testicular dysgenesis syndrome (TDS) and represents one of the models that permit the description of the pathogenesis of testicular cancer, being associated with this malignancy in over 25% of cases [150,151]. TDS is undoubtedly a multifactorial disease with established causes of severe forms being primarily genetic (i.e., SRY mutation, androgen insensitivity). Nevertheless, the significant increase in testicular cancer in the last years cannot be explained by genetic factors alone and environmental and lifestyle factors have to be considered both for testicular cancer and TDS [152,153]. Furthermore, due to the consideration of the relation between age, androgens, and testicular cancer, the presence of hormonal alterations as well exposure to endocrine-disrupting compounds could represent, in addition to hereditary predisposition and in utero alterations, another side of the coin, due to the potential second hit on testis carcinogenesis processes [129].

## 7. Conclusions and Future Perspectives

Current information on the causes of testicular cancer as well as risk factors for this disease is still limited, and even if the aim of this review was to summarize global data on testicular risk factors, most of the available data are geographically limited to the United States and Northern Europe, especially when considering occupational risk factors. Although a large piece of knowledge suggests that most testicular cancers originate from a potential noxa during fetal life, the role of the environment, familial history, ethnicity as well as diet and occupational exposures are a few of the actors involved in testis carcinogenesis. The relative rarity of the condition has limited the possibility to perform large linkage studies as well as investigate several modifiable factors such as drugs and diet [89,154]. Considering that testicular cancer is one of the most curable types of cancer and the high survival rate, improvement in the early diagnosis could further limit the impact on the health and fertility of this disease. Despite the increasing technological advancement in diagnostic tools and techniques, with the consolidation of the role of liquid biopsy and imaging in most urological cancers including testicular cancer, the identification of risk factors associated with testis carcinogenesis could further improve the chance of proper treatment and early diagnosis, permitting to intercept possible populations or individuals at risk, tailoring appropriate screening and surveillance measures [155,156,157,158]. Currently, the main limitation to the identification of probable and unknown risk factors related to testicular cancer is related to the relative rarity of the conditions which could have not permitted the enrollment of large cohorts for epidemiologic and linkage studies. Similarly, the studies analyzing the genetic risk factors suffer from the same limitations, due to the difficulties in obtaining sufficient data regarding the lineage of families affected by this disease. However, the possibility to organize countries and even continent-related studies could provide further insight into the epidemiologic risk factors linked to testicular cancer. Nevertheless, while the identification of these risk factors would be pivotal in further understanding the disease, several limitations have to be reported. Firstly, some risk factors traditionally associated with testicular cancer are still not well understood in terms of pathogenesis (i.e., cryptorchidism and age), making it difficult to accurately quantify their impact on the development of the disease. Additionally, other risk factors may be influenced by confounding variables, making it challenging to determine their true relationship with testicular cancer (i.e., familial history, age at puberty, infections). Another limitation is instead related to the fact that not all individuals with known risk factors for testicular cancer will actually develop the disease, highlighting the need for further research aimed at assessing the underlying mechanism which contributes to testis carcinogenesis. Lastly, the identification of risk factors alone could not predict high-risk individuals as many cases occur in individuals with no known risk factors [159,160]. A comprehensive approach that takes into account both epidemiologic and genetic risk factors is necessary to accurately assess individual risks of developing testicular cancer and, therefore, tailoring a proper screening.

## Figures and Tables

**Table 1:** Known risk factors associated with testicular cancer.

Known Risk Factors	OR	Reference
Cryptorchidism	3.7–7.5	[26,27]
First-degree relatives of patients with testicular cancer	3.1	[31]
Brother with testicular cancer	6.3	[35]
Father with testicular cancer	4.4	[35]
Low birth weight	1.34	[39]
Exposition to diethylstilbelstrol during pregnancy	2.98	[42]
Height (every 5 cm increase)	1.13	[62]

**Table 2:** Probable risk factors associated with testicular cancer.

Risk Factor	Limitations	Reference
Caucasian ethnicity	Socio-economic and environmental exposure disparities	[49,50]
HPV, HIV	Marked heterogeneity	[67]
Epididymoorchitis	Limited geographical area of the study, lack of bacterial culture and data regarding environmental exposure	[68]
Smoking	Several confounding factors	[75,76]
Cannabis use	Rationale still unclear	[80,81]
High consumption of dairy products, red meat and baked products	Limited reliability of data acquired via questionnaires	[89,90,91]
Exposition to electromagnetic fields	Few studies, case reports	[100,101]
Occupational exposure to plastics and high temperatures	High heterogeneity, biased data	[104,105,106,107,108]
Occupation as firefighter and military personnel	Hypothesized exposition to endocrine disrupting factors	[108,117,118,121,122,123]

## Data Availability

Data analyzed in this study were a re-analysis of existing data, which are openly available at locations cited in the reference section.
